# Important Topics in the Future of Tissue Engineering: Comments from the participants of the 5th International Conference on Tissue Engineering at Kos, Greece

**DOI:** 10.1093/rb/rbu001

**Published:** 2014-10-20

**Authors:** Fu-Zhai Cui, Antonios G. Mikos

**Affiliations:** ^1^Institute for Regenerative Medical Materials, School of Materials Science and Engineering, Tsinghua University, Beijing 100084, China; ^2^Department of Bioengineering, Rice University, Houston TX77005, USA

Over 150 participants from around the world congregated on the beautiful island of Kos—home of Hippocrates, Father of Medicine—from June 20 to June 25, 2014, to share their research findings and engage in dialogue pertaining to progress in tissue engineering. Since the field’s inception, we have witnessed its astonishing development over the past 30 years and the groundbreaking work stemming from both laboratory and clinical settings. Despite continuously evolving concepts and strategies, the essential ingredients of scaffolds, cells and growth factors remain to be explored further. While stem cells presently pique tremendous interest within the research community, they have yet to come to the mainstream in the clinical context, as current regulated medical devices predominantly involve only scaffolds and several growth factors. In light of this bench-to-bedside disparity, participants should evaluate the translational aspects of their work and how it can be incorporated into the future of tissue engineering and regenerative medicine. Furthermore, researchers may consider expanding their focus to include currently untapped technologies with potential to propel the field forward.

The 5th International Conference on Tissue Engineering provided a global forum for established leaders and emerging young scientists in the field, to showcase their diverse ideas on advanced tissue engineering and regenerative medicine. In particular, new biomaterial-based strategies were proposed to improve the quality of engineered tissue, to increase spatiotemporal release of growth factors, and to elucidate and modulate cell signaling pathways. Improvements in scaffold fabrication methodologies, cell delivery approaches and cell differentiation protocols were also discussed. The topical breadth was further demonstrated through presentations on imaging modalities and computational modeling. Overall, this conference provided a comprehensive, enriching overview of the field and alluded to trends in the years to come.

In what follows, we present a commentary from nine participants who are active professors representing various branches of tissue engineering. We hope that their insight may stimulate further discussion by the readers.

## Tony Weiss


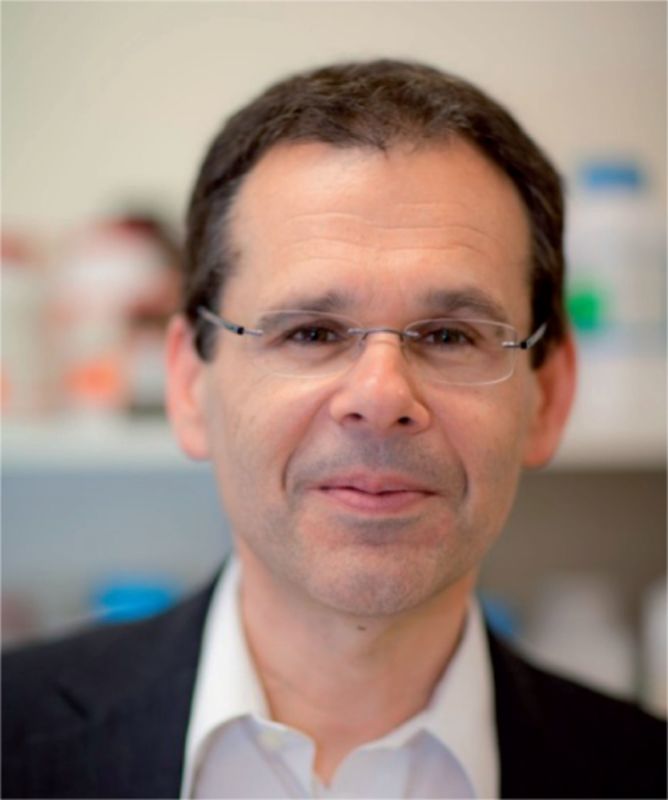


Professor of Biochemistry and Molecular Biotechnology at the University of Sydney, Professor at the Bosch Institute, Professor at the Charles Perkins Centre, Professor at the Royal Prince Alfred Hospital (Honorary), Distinguished Visiting Professor at Brain Korea 21 and founder of a biotechnology company. He is the Chair of the Matrix Biology Society of Australia and New Zealand and elected to the regional Tissue Engineering and Regenerative Society Council. In addition, he has been appointed to several national organizations including the Australian Biotechnology Advisory Council, National Enabling Technology Strategy Advisory Council, and Biological Sciences and Biotechnology, Australian Research Council College of Experts where he held the national Chair. He is an inventor with 23 awarded international patents to his credit. The Weiss Laboratory at the University of Sydney is the leading research site for tropoelastin and synthetic elastin biomaterials.

***Comment*:** *There are many fundamentally important topics in tissue engineering, so it is not possible to single out just one area. However, in my research focus, which is elastic tissue augmentation and repair, I believe that the major drive will be towards integrating increasingly sophisticated architectures and cellular signals. That will help us harmonize accelerated repair and integration to give true four-dimensionality: constructs that accommodate the three dimensions of functional systems and are combined with real-time responses over extended lifespans.*

## Warren Grayson


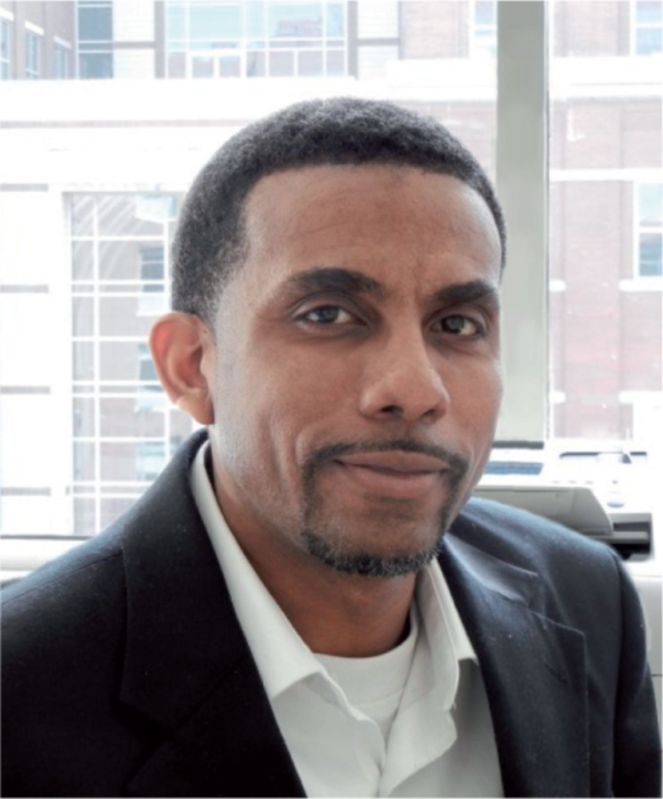


Assistant Professor of Biomedical Engineering and Material Sciences and Engineering at Johns Hopkins University and is a founding member of the Translational Tissue Engineering Center. He obtained his Ph.D. in Biomedical Engineering from Florida State University, and completed his postdoctoral training at Columbia University in New York. He has authored over 40 original and review articles and book chapters, and holds several patents for bioreactor and biomaterial designs. Currently, his laboratory focuses on spatial and temporal regulation of stem cell differentiation in 3D constructs to generate clinically useful engineered grafts.

***Comment*:** *Over the past 30 years, the burgeoning field of tissue engineering (TE) has seen significant advances in the development of new biomaterials and the application of stem cells to tissue regeneration. In spite of these myriad accomplishments, we have seen relatively few TE products commercialized and/or widely used clinically. Therefore, there is a huge impetus for the development of TE approaches that can overcome regulatory hurdles to be translated to the clinic and also have the potential to be commercially viable. ‘Smart’ biomaterials that guide the in situ development of endogenous or transplanted cell populations into functional tissues offer tremendous potential as off-the-shelf therapies. Consequently, future studies should examine how these materials interact with readily procurable cell populations, such as adult stem cells or induced pluripotent stem cells (iPSCs). Hitherto, there are several examples of the stem cell applications in clinical trials. Effectively combining these cell populations with smart biomaterials could revolutionize current treatment methods for a number of debilitating injuries and diseases and deliver on the therapeutic promise of TE.*

## Jan de Boer


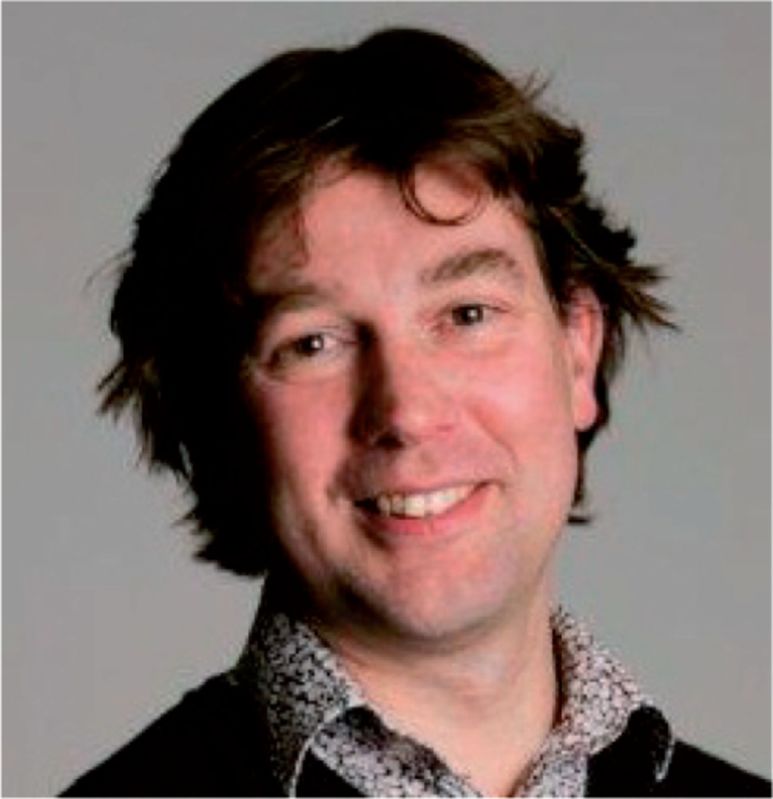


Professor of Applied Cell Biology at the Merln Institute, University of Maastricht, The Netherlands. He obtained his Ph.D. at the Erasmus MC – Rotterdam in 1999, after which he worked as a postdoc in Cambridge, UK. In 2002, he started as a research associate at IsoTis B.V. in Bilthoven. In December 2003, de Boer was appointed Associate Professor at the University of Twente. After a 12-month sabbatical at the Wyss Institute and the Broad Institute of MIT and Harvard, he became Full Professor and Chair of the Department for Cell Biology-Inspired Tissue Engineering (cBITE) at the Merln Institute in 2014.

***Comment*: ***In these days of big data, I see a bright future for holistic biomaterials research, which we term* materiomics*. Biomaterials come in many shapes and forms. Their design space is enormous because many parameters can be changed such as bulk chemistry, surface topography, 3D geometry, degradation properties, etc. Efficiently maneuvering through and exploring all these possibilities, for instance using modeling and high-throughput screening, is a major challenge. On the other side of the interface, the molecular mechanisms guiding the response of cells to materials is largely undefined. Here, genomics-based strategies can assist in untying the knot. Both in materiomics and genomics, efficient generation and mining of data is the key, and I foresee an increasing role for bioinformatics and computational modeling in the field of biomaterials research.*

## Guillermo Ameer


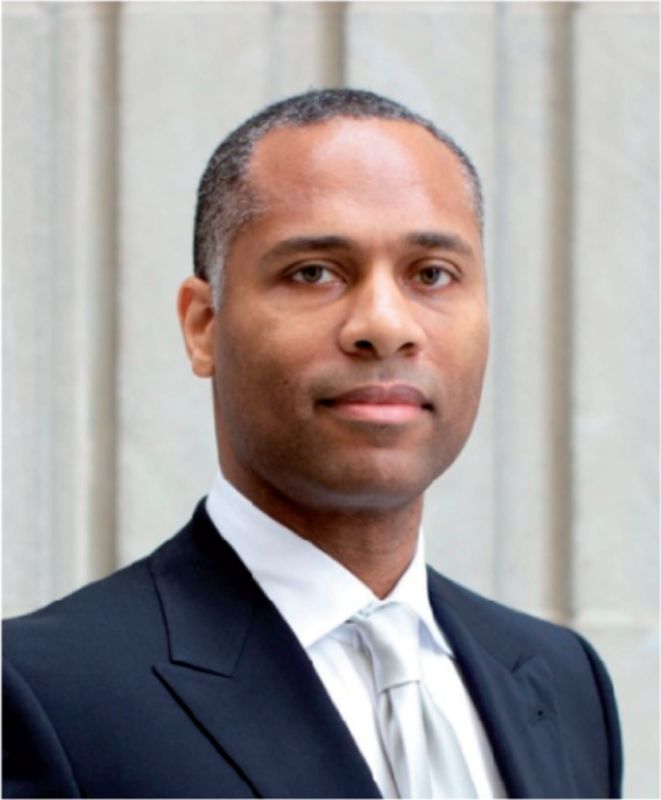


Dr Ameer is a Professor in the Biomedical Engineering Department at the McCormick School of Engineering and the Department of Surgery at the Feinberg School of Medicine, Northwestern University. He is also a resident faculty at the Institute for BioNanotechnology in Medicine and a member of the Chemistry of Life Processes Institute. His research interests include biomaterials, vascular and orthopaedic tissue engineering, regenerative medicine, controlled drug delivery and bio/nanotechnology for improved therapeutics and diagnostics. Specifically, Dr Ameer’s laboratory pioneered the development of citric acid-based polyesters, referred to as polydiolcitrates. He has co-authored over 100 peer-reviewed journal publications and conference abstracts, several book chapters, and over 25 issued and pending patents. Dr Ameer has received numerous awards, including election to *Technology Review* magazine’s top 100 Young Innovators in the world, the NSF CAREER award and the American Heart Association’s Established Investigator Award. He has served on several national and international scientific review committees for funding research. He is a fellow of the American Institute of Medical and Biological Engineering and served as a permanent member of the Musculoskeletal Tissue Engineering study section of the National Institutes of Health. Dr Ameer is on the editorial boards of the *Journal of Biomedical Materials Research: Part A and Organogenesis*. He was the co-founder of several medical device companies in the areas of dialysis, vascular surgery, orthopedic surgery, and wound care management.

***Comment*:** *An important topic for tissue engineering will be the development of strategies to promote and monitor the integration of engineered tissue or devices such as scaffolds that carry cells or other biological factors with host tissue. To this end, the development of biomaterials and devices that can interact in a positive way with the ensuing inflammatory response to the implant and also potentially report on its status (or surrounding environment) will be critical. In addition, oxidative stress is a major component of the inflammatory response that is often overlooked in tissue engineering. Therefore, obtaining a better understanding of how biomaterials contribute to oxidative stress either through degradation products or perturbing the redox state of adjacent cells will be important to controlling the body’s response to injury, the scaffold device or the engineered tissue.*

## John A. Jansen


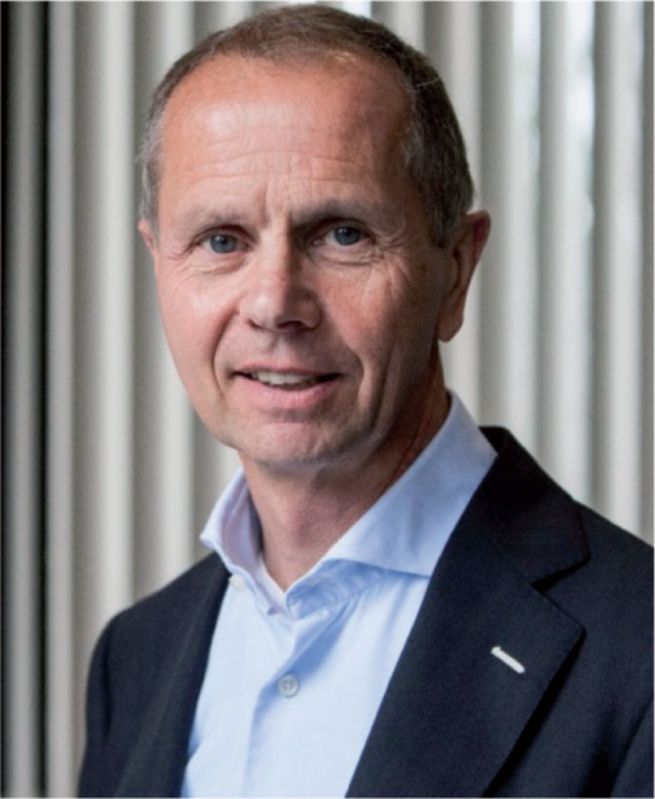


John A. Jansen studied Dentistry at Radboud University Nijmegen and graduated in 1977. He completed his Ph.D. in 1984 at the Radboud University Nijmegen. After working in Amsterdam and Leiden he returned to Nijmegen in 1991 to become Associate Professor of Biomaterials before being appointed Full Professor in April 1996. In April 2008, he was elected as full member of the Royal Netherlands Academy of Arts and Sciences. Additional awards include: PIONEER Award Netherlands Organization for Scientific Research (1991), Simon Stevin Mastership of the Netherlands Technology Foundation (2003), Clemson Award for Outstanding Contributions to the Literature of the Society for Biomaterials (2004), Federa-award of the Federation of Dutch Medical Scientific Societies (2012), Knight in the Order of the Netherlands Lion by King Willem-Alexander of the Netherlands, Isaac Schour Award International Association of Dental Research (2014). He has contributed to over 560 publications, is the owner of 7 patents and is an editorial board member/editor of 8 international scientific journals.

***Comment*:** *Over the last decades, pre-existing biomaterials have been re-designed from an engineering attitude for use in regenerative medicine. However, the resulting range of relatively inert biomaterials exhibit disappointingly limited long-term clinical success, since they lack the biological and mechanical functionality of natural tissues. As a consequence, these passive biomaterials have a limited capacity to interact with the physiological environment and induce tissue regeneration. The implantation of a biomaterial and its subsequent tissue integration requires symbiosis between a biological and a non-biological system. Recently, it has been recognized that a bio-inspired design of biomaterials holds strong promise to construct a new generation of adaptive implant materials that are able to provide effective biological cues to the physiological environment and instruct stem cells toward predefined lineages. In a natural environment, the cells of our body interact with the extracellular matrix (ECM) that is surrounding them. However, the ECM is not just an inert scaffold, but a dynamic system that is impacted by the presence of cells providing in turn reciprocal molecular and mechanical feedback to cells. Thereby, the ECM strongly influences cellular behavior in terms of adhesion, migration, proliferation and differentiation. When examined from a topographical point of view, it becomes apparent that this ECM is structured as a mixture of pores, ridges and fibers, which all are sized in the nanoscale range. Breakthroughs in nanoscience and nanotechnology provide tools to structure molecules and biomaterials at the nanometer scale and to exploit the unique properties of nanomaterials. It is vital to understand that nanomaterials have physicochemical and biological properties that can be totally different from their ‘macroscopic’ counterparts. Therefore, bio-inspired nano-biomaterials are the future for tissue regenerative purposes.*

## Yadong Wang


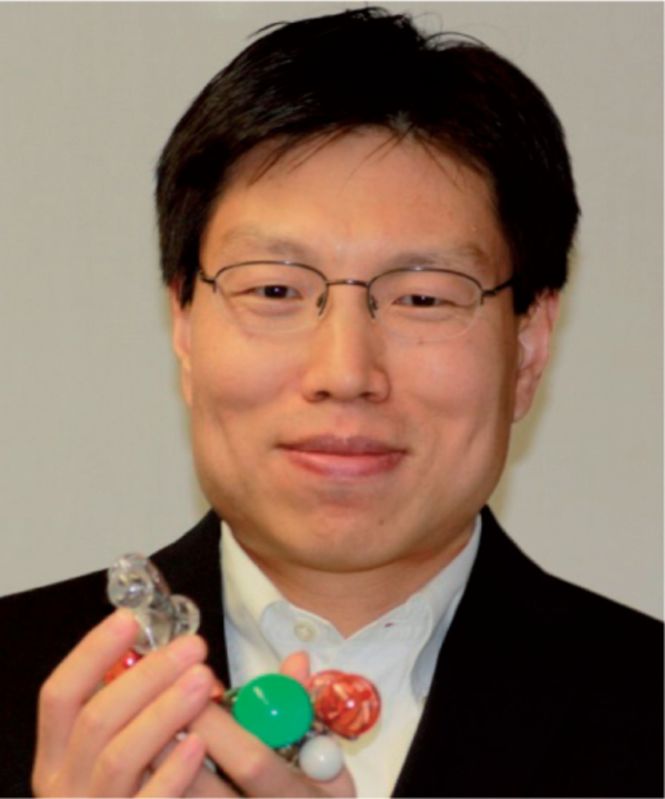


Yadong Wang is the William Kepler Whiteford Professor in Bioengineering with adjunct positions in Chemical Engineering, Surgery, and Mechanical Engineering and Materials Science at the University of Pittsburgh. He obtained his Ph.D. degree in Chemistry at Stanford University in 1999, and performed his postdoctoral studies at MIT. He joined the Bioengineering Department at the University of Pittsburgh in 2008 after serving as an Assistant Professor at the Georgia Institute of Technology for 5 years. He has published articles in journals including *Science*, *Nature* research journals and *PNAS*.

***Comment*:** *There are many challenges in tissue engineering, and correspondingly there are many important topics. Among the myriad approaches to tissue engineering,* in situ *tissue engineering is experiencing a renaissance. As it relies on the host as cell source and bioreactor and bypasses cell seeding and cell culture steps,* in situ *tissue engineering is likely the fastest way to translate laboratory discoveries to clinical benefits. Within the framework of* in situ *tissue engineering, there are three important topics: (i) understand and modulate the host inflammatory response to biomaterials; (ii) invent effective means to recruit the desired cell types to the implant; and (iii) design materials that will induce the recruited cells to synthesize appropriate extracellular matrix including maturation of the matrix molecules.*

## Shengmin Zhang


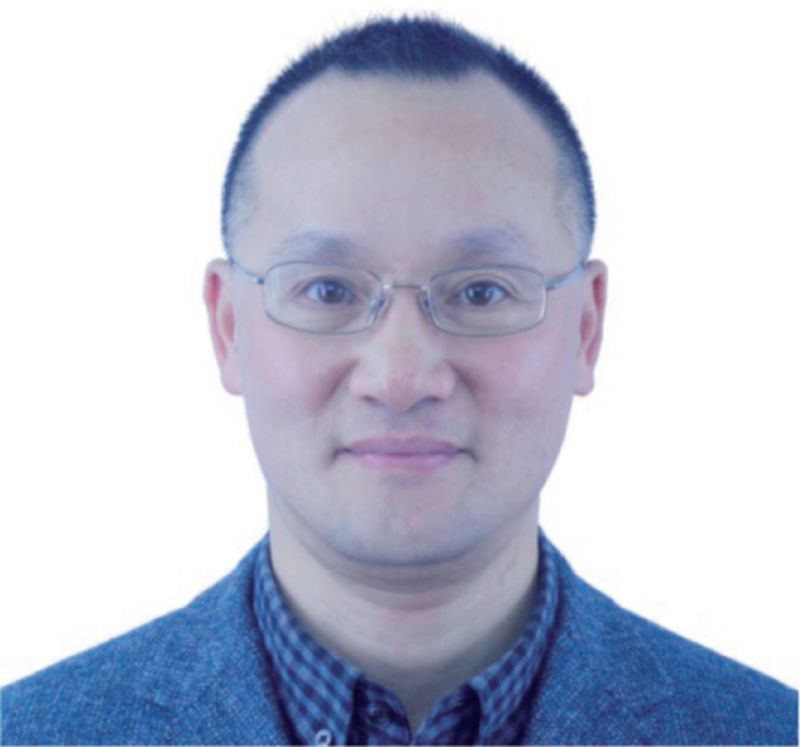


Dr Shengmin Zhang is the Chair Professor and the Director of the Advanced Biomaterials and Tissue Engineering Center at Huazhong University of Science and Technology, China and the Founding Chair of the China-Korea Center for Biomaterials and Nano-Biotechnology. He received his Ph.D. in biomedical materials from Wuhan University of Technology. He has co-authored more than 100 peer-reviewed papers, 5 books and has given about 50 keynote or invited speeches in various conferences. He is also the inventor of more than 30 patents, which have led to 2 Product Certificates of Registration authorized by CFDA. His research interests focus on frontier on biomaterials and medical devices, like 3D bioprinting and biofabrication, interaction among materials, cells/tissue and microenvironments.

***Comment*:** *Biomaterials and tissue engineering are on the eve of a profound revolution. An obvious trend for tissue engineering is moving from an* in vitro *one into an* in vivo *one. Biomaterials and scaffolds are developed from those with biological growth factors (GFs) to those of the free GFs which not only return to their original nature, but are also easier to meet the requirements of FDA regulations and clinical applications. Of course, such changes are challenging the design and fabrication of biomaterials more than ever. Presently, biomaterials and scaffolds are expected to have functions with* in vivo *homing cells and GFs, and then releasing these GFs sequentially. Future biomaterials based on new concepts and principles will be designed at the molecular level to be* in situ *utilized by cells and tissue to accelerate reconstruction and regeneration, rather than to only be degraded into waste. In addition, special attention should be paid to those new emerging tissue regenerative materials with micro-/nano-structures and surface 3D topography, which could stimulate specific biological responses to cells, tissue and* in vivo *microenvironments.*

## Dongqing Wang


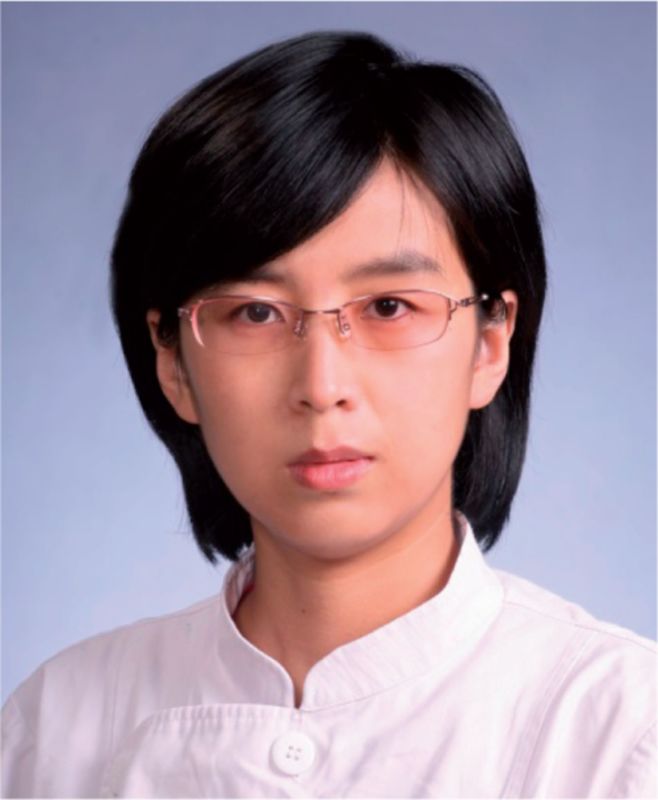


Associate Professor of Periodontology at Beijing Stomological Hospital, Capital Medical University and the Committee Members of Chinese Society of Periodontology. Dr Wang graduated from Dental College, Hebei Medical University in 1997, and got her Ph.D. from Tokyo Medical and Dental University in 2005. Her research is mainly on periodontitis with cardiovascular diseases, laser in periodontitis and peri-implantitis, and periodontal regeneration.

***Comment*:** *The next decade would present us more challenges and exciting research topics in the rapidly developing field of tissue engineering. Emphasis is still placed on the optimal cell source, growth factors, scaffold design and fabrication, and the development of microfabrication technology to create vascularized tissues and organs. My research interest is on periodontal regeneration. A major challenge is the microbial contamination that plagues periodontal wound microenvironment. It is of great significance that one biomaterial could enhance tissue growth and diminish bacterial function. From surgical point, injectable biomaterial combined with growth factor will be a good means. In addition, current regenerative options are limited in intrabony defects. In fact, there is no effective regenerative strategy for horizontal bone loss. In the near future, stem cell therapy, cell sheet engineering and growth factor with novel biomaterial scaffold will be useful for the regeneration of lost tissue.*

